# Effects of temperature on gene expression in embryos of the coral *Montastraea faveolata*

**DOI:** 10.1186/1471-2164-10-627

**Published:** 2009-12-23

**Authors:** Christian R Voolstra, Julia Schnetzer, Leonid Peshkin, Carly J Randall, Alina M Szmant, Mónica Medina

**Affiliations:** 1 Red Sea Research Center, King Abdullah University of Science and Technology (KAUST), Thuwal, Saudi Arabia; 2 School of Natural Sciences, University of California, Merced, 5200 North Lake Road Merced, CA 95343, USA; 3 Systems Biology, Harvard Medical School, 200 Longwood Ave, W Alperts #536, Boston, MA 02115, USA; 4 Center for Marine Science, University North Carolina Wilmington, 5600 Marvin K Moss Lane, Wilmington, NC 28409, USA

## Abstract

**Background:**

Coral reefs are expected to be severely impacted by rising seawater temperatures associated with climate change. This study used cDNA microarrays to investigate transcriptional effects of thermal stress in embryos of the coral *Montastraea faveolata*. Embryos were exposed to 27.5°C, 29.0°C, and 31.5°C directly after fertilization. Differences in gene expression were measured after 12 and 48 hours.

**Results:**

Analysis of differentially expressed genes indicated that increased temperatures may lead to oxidative stress, apoptosis, and a structural reconfiguration of the cytoskeletal network. Metabolic processes were downregulated, and the action of histones and zinc finger-containing proteins may have played a role in the long-term regulation upon heat stress.

**Conclusions:**

Embryos responded differently depending on exposure time and temperature level. Embryos showed expression of stress-related genes already at a temperature of 29.0°C, but seemed to be able to counteract the initial response over time. By contrast, embryos at 31.5°C displayed continuous expression of stress genes. The genes that played a role in the response to elevated temperatures consisted of both highly conserved and coral-specific genes. These genes might serve as a basis for research into coral-specific adaptations to stress responses and global climate change.

## Background

Coral reefs are based on the symbiotic relationship between corals and photosynthetic dinoflagellates of the genus *Symbiodinium*, also known as zooxanthellae, and exist within a narrow temperature range. The optimum temperature for adult scleractinian corals is between 25°C and 29.0°C [1]. As climate change becomes an increasing threat to the biosphere, corals are among the first organisms to suffer from the consequences of global warming [2]. Heat stress in reef-building corals affects the coral hosts and their algal symbionts, but the relative sensitivity of both to thermal stress is uncertain [3]. The first visual sign of heat stress to the coral holobiont (i.e. host, symbionts, and associated microorganisms) is bleaching, i.e. the loss of photosynthetic symbionts [4].

Studies on heat stress in adult corals have shown that processes such as Ca^2+ ^homeostasis, cytoskeletal organization, cell death, calcification, metabolism, protein synthesis, and heat shock protein activity are affected among others [5-9]. Many of the identified genes from these studies code for known stress-responsive proteins that are shared among eukaryotes. Furthermore, it has been shown that an increase in temperature leads to oxidative stress in corals [10], with evidence pointing towards photosystem II of the algal symbiont as the main source of reactive oxygen species (ROS) [11-13].

Larvae play an important role in coral reef ecosystems as they form the starting point of the bentho-pelagic lifecycle of a coral [14]. From a molecular and genetic perspective, coral embryos/larvae represent an interesting system, as many species initially lack endosymbionts. Hence, it is possible to measure the effect of temperature on corals without the confounding factor of symbionts and their different physiologies. Studies on coral larvae show that increasing temperatures affect fertilization, embryogenesis, development, survival, and settlement [15,16]. However, molecular studies that assess transcriptome-wide changes in gene expression upon increasing temperatures in coral embryos and larvae have not yet been published.

In this study, we exposed newly fertilized azooxanthellate coral embryos of the Caribbean species *Montastraea faveolata *(Cnidaria, Anthozoa, Hexacorallia) to a range of temperatures: 1) a permissive temperature of 27.5°C that is known to be non-stressful; 2) 29.0°C, which is a normal summer seawater temperature in the Caribbean Sea during the spawning period; and 3) an elevated temperature of 31.5°C, which has been observed during the late summer of bleaching years such as 2005. Transcriptomic changes were assayed with microarrays at 12 and 48 hours after fertilization. Based on our analysis of differentially expressed genes we devised a model that proposes that genes that play a role in system perturbation, system maintenance, and system regulation are affected upon heat stress. This study is the first transcriptome-wide analysis of heat stress in coral embryos and our data provide first insights into the relevant genes and adaptive capabilities of coral embryos in light of projected increases in seawater temperatures.

## Results and Discussion

### Developmental differences

We compared developing coral embryos after 16 and 50.5 hours that were raised at 27.5°C, 29.0°C, and 31.5°C (Table 1). Albeit offset by 4 and 2.5 hours to the samples on the microarray, the developmental stages of these embryos should reflect the embryos that were used for microarray analysis. We were not able to identify developmental differences based on larval morphology in regard to the different temperatures. However, tools for external classification of developmental stages are limited. Hence, changes in development probably exist that are not detected morphologically, but confound gene expression nevertheless. Studies have shown that some coral species do show faster development upon increasing temperatures, whereas others do not [15,16].

**Table 1:** Developmental stages of coral embryos after 16 and 50.5 hours raised at 27.5°C, 29.0°C, and 31.5°C

		Number of embryos in that stage				
		
Temperature	Hours after Fertilization	Blastula	Invaginated	Gastrula	Planula	Irregular
27.5°C	16	98	0	0	0	3
29.0°C	16	88	3	0	0	9
31.5°C	16	98	2	0	0	9

27.5°C	50.5	0	0	88	1	11
29.0°C	50.5	0	0	96	0	4
31.5°C	50.5	0	0	97	0	4

We found a higher number of misshapen embryos after 12 hours at 29.0°C and 31.5°C in comparison to embryos kept at 27.5°C. However, after 48 hours, the proportion of misshapen embryos decreased for embryos kept at 29.0°C and 31.5°C, and increased for embryos kept at 27.5°C. Negri *et al*. [15] observed an increase in frequency of abnormalities in embryos of *Acropora millepora *when exposed to temperatures of 32°C or higher, but in our case the number of misshapen embryos was not consistent with an increase in temperature. However, proper larval development is critical for larval settlement, which in turn is required for individuals to be incorporated into a population. We have not followed up development of larvae until settlement, but the number of misshapen embryos indicates that it is a confounding factor to gene expression measurements.

### Embryo transcriptomes are affected in a time- and temperature-dependent manner

**Figure 1:**
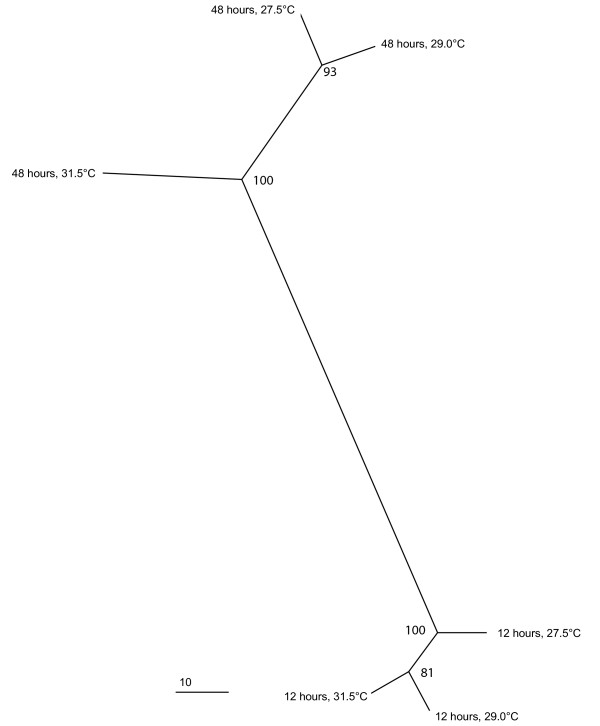
**Radial tree of hierarchically clustered transcriptomes of embryos of *M. faveolata***. Embryos were raised at three different temperatures (27.5°C, 29.0°C, 31.5°C), and gene expression was measured at 12 and 48 hours after fertilization. Support values are based on 1,000 bootstrap replicates. Scale bar at lower left corner displays overall Euclidean distance between expression vectors.

We constructed a radial tree of hierarchically clustered gene expression data to infer relatedness across different temperature regimes and time points (Figure 1). We found that samples clustered according to time point, i.e. all 12 hour samples were distant from all 48 hour samples. Hence, the differences between all temperature treatments for a given time point were much smaller than between temperatures for both time points. We suggest that the stark separation between both time points is attributable to the different developmental stages the embryos were in (Table 1) rather than differences due to temperature treatment, which is why we decided to not compare directly between time points. A recent study by Grasso *et al*. [17] found that 1,084 of 5,081 unique genes were differentially expressed during early coral development. Thus, a significant part of the coral embryo transcriptome seems to change during development, which supports the distinctive separation we see between 12 and 48 hour old embryos.

For the 12 hour time point, embryos kept at 29.0°C and 31.5°C cluster together and are separated from embryos kept at 27.5°C. We interpret this pattern as an indication that embryos initially respond similarly to increased temperatures. However, the distance between all 12 hour nodes is small. By contrast, after 48 hours embryos kept at 27.5°C cluster together with embryos kept at 29.0°C and are notably separated from embryos kept at 31.5°C. This indicates that over time the transcriptomic pattern of embryos kept at 29.0°C becomes more similar to those raised at 27.5°C.

### Molecular signature of temperature increase in coral embryos

We identified a total of 232 differentially expressed genes for embryos kept at 29.0°C after 12 hours post fertilization, and 101 differentially expressed genes for embryos at this temperature after 48 hours. For embryos kept at 31.5°C, we identified 218 differentially expressed genes after 12 hours, and 285 after 48 hours (Additional File 1). Genes were defined as up- or downregulated in regard to the expression of that gene in the 27.5°C temperature sample. Please note that the fertilization temperature was 30°C, and thus higher than two of the experimental temperatures (27.5°C and 29.0°C) and lower than the third (31.5°C). Gene expression might be partially affected by this circumstance as larvae at 27.5°C and 29°C experienced a higher initial incubation temperature, and larvae at 31.5°C experienced a lower initial incubation temperature. This might especially affect the 12 hour time point as larvae were kept at the treatment temperatures for only 10 hours. Annotated differentially expressed genes were assorted manually into different categories depending on their function or process they are involved in (based on BLAST, HMMER, GO, and InterPro results). Genes were associated with the following categories: response to stress, response to oxidative stress, apoptosis, immune system, cytoskeleton, proliferation/growth/development, ribosomes/translation, degradation, metabolism, electron transport, transport, signalling, RNA, DNA, and regulation of transcription (Additional File 2). These categories were then assembled into the three main groups of (1) system perturbation, (2) system maintenance, and (3) system regulation. The system perturbation group contained all the genes that became differentially expressed as a direct consequence of temperature exposure, i.e. genes assorted to a response to stress, a response to oxidative stress, apoptosis, cytoskeleton, and the immune system. Genes that were assorted to cytoskeleton, proliferation/growth/development, ribosomes/translation, degradation, metabolism, electron transport, and transport were united into the group system maintenance. This group contained all those genes that play roles in housekeeping processes or processes related to continuation of the biology of a cell. All genes that were assorted to signaling, RNA, DNA, and regulation of transcription were referenced in the group system regulation as these proteins play roles in regulatory processes in a cell.

### System perturbation genes

We identified stress-responsive genes such as Stress-response protein NST1 (AOSB460), Universal stress protein (AOSF1521), Drought-induced protein RDI (CAON1066), Recombination repair protein 1 (AOSB392), and Senescence-associated protein (CAOO2479, CAOO703) among others. We found a consistent down-regulation of members of the SCRiPs gene family across all time points and temperatures analyzed. This gene family has only been recently identified [18], and has been shown to be downregulated upon thermally induced bleaching in *M. faveolata *[6].

We did not identify a significant upregulation of heat shock proteins (HSPs) at any time point and temperature. This could be due to a maximum temperature increase of up to 31.5°C. Most studies on proteins have not reported the expression of heat shock proteins at temperatures lower than 33°C [5,8]. In addition, our arrays contained only homologs of a 90-kda heat shock protein (HSP90) (AOSF1451) and a 97 kDa heat shock protein (CAOO2018). Both genes displayed higher expression in embryos kept at 31.5°C after 12 hours, but not significantly so (data not shown). We did, however, identify heat shock transcription factor 1 (CAON1605) to be upregulated after 12 hours at 31.5°C. Heat shock transcription factors (HSFs) regulate the induction of many HSPs and other proteins [19,20]. In particular, HSF1 has been indentified as the primary transcription factor responsible for the transcriptional response to heat stress in mammalian cells [21]. In addition, our set of differentially expressed genes indicated increased rates of protein misfolding, degradation, and DNA damage as stated in succeeding sections. Hence, we assume that the treatment temperatures applied were high enough to have structural effects on nucleic acids and proteins. This in turn suggests that both temperatures, 29.0°C and 31.5°C, are stressful, and that rather the buffering capacity of heat shock proteins was being compromised in this experiment.

In the category response to oxidative stress we found a high overlap among the upregulated genes after 48 hours for embryos kept at 29.0°C and 31.5°C. At both temperatures cytochrome p450 (CAON1879), soma ferritin (CAON1101), catalase (AOSF550), and peroxidasin-like protein (AOSF997) were upregulated as a consequence of exposure to increased temperatures. The upregulation of oxidative stress genes indicates that, as in adult corals, an increase in temperature leads to oxidative stress in coral embryos [11]. Whereas cytochrome p450, soma ferritin, and catalase displayed a similar change in expression at both temperatures, peroxidasin-like protein was highly upregulated (> 12-fold) in embryos exposed to 31.5°C. Peroxidasin is a supposedly multifunctional protein that plays a role in several biological processes such as oxidation reduction, removal of apoptotic cells, and cross-linking and stabilizing of the extracellular matrix [22]. We suggest that peroxidasin-like protein might provide a useful candidate as a coral heat stress biomarker for the following reasons: 1) Peroxidasin-like protein seems to be concordantly upregulated in embryos exposed for prolonged periods of time to elevated temperatures, 2) it showed a difference in upregulation depending on the temperature level that embryos were exposed to which gives rise to the possibility to not only assess current stress levels, but past stress levels, and 3) it has been shown to be upregulated upon heat stress in adult coral [6]. Further studies on peroxidasin-like protein with a higher number of samples and coral species must be conducted in order to yield insights into the identity and applicability of this protein as a heat stress biomarker.

We find evidence for activation of apoptosis upon exposure to increased temperatures. We identified a homolog of activating transcription factor 5 (CAON612) to be upregulated after 12 hours for both temperatures, and after 48 hours for embryos raised at 31.5°C. This gene plays an essential role in cell growth, survival and apoptosis [23,24]. Moreover, we identified a number of downregulated apoptosis-related genes, mainly in the late time point. The genes were autophagy-related protein 16 (CAOO2671), calreticulin (AOSC448), and Proapoptotic Caspase Adapter Protein (AOSF761). Downregulation of calreticulin has been shown to activate oxidative stress and cell death [25]. Similarly, downregulation of proapoptotic caspase adapter protein (PACAP) has been shown to expose neuronal cells to oxidative stress-induced apoptosis [26-28].

### System maintenance genes

Differential expression in developmental- and growth-related genes could be a consequence of differences in development for a given time point. For this reason, we decided that the implication of differentially expressed genes of this category remains to be elucidated. Nevertheless, downregulation of calmodulin (AOSF573) has been shown to be a sign of oxidative stress [29]. Furthermore, we detected downregulation of ribosomal and translation-associated proteins. Downregulation of ribosomal proteins as a consequence of heat shock has been shown in *Drosophila melanogaster *and yeast [30,31]. Most of the genes in the category degradation were ubiquitin-related genes. Protein degradation is considered a general consequence of heat stress as elevated temperatures lead to increased rates of misfolding. In accordance with our findings of a downregulation of protein biosynthesis (which would indicate a downregulation of overall metabolism), we found a stronger down- than upregulation of metabolism-related genes. We also found a downregulation of genes related to electron transport in oxidative phosphorylation that might further indicate that metabolism is downregulated. For instance, cytochrome b5 (CAON1668) and NADH-ubiquinone oxidoreductase 7 (CAON943) were downregulated across all time points and temperatures, mitochondrial ATP synthase f chain (AOSC720) in both time points at 29.0°C and in the late time point at 31.5°C.

### System regulation genes

We found a homolog of histone H3.3 consistently downregulated across all treatments and time points, and a homolog of histone H2A.V downregulated after 48 hours for both treatment temperatures. Histone proteins are post-translationally modified and are part of chromatin-based regulatory mechanisms that modulate the accessibility of genetic information [32]. It remains to be determined what the direct consequences of histone downregulation in temperature-treated coral embryos are, but it has been shown that downregulation of histone gene expression is used as a mechanism to prevent cells from further replicating upon DNA damage [33].

A number of upregulated genes contained zinc finger motifs. Zinc finger proteins comprise the largest family of regulatory proteins in mammals and bind to cognate DNA (e.g. transcription factors), RNA, or protein [34]. Specifically proteins that use C2H2 zinc fingers have been found to recognize histones for acetylation and/or methylation [35,36]. Although tempting, it remains to be determined if the histones and zinc finger genes we identified here are functionally connected.

### Model of heat stress in coral embryos

**Figure 2:**
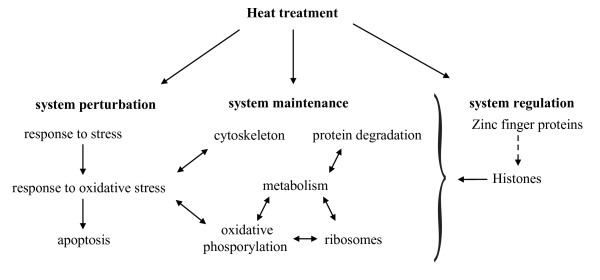
**Model of heat stress response in coral embryos**. Upon heat treatment, coral embryos respond with regulation of genes playing a role in system perturbation, system maintenance, and system regulation. These functional groups are interconnected.

Based on our analysis and assignment of annotated differentially expressed genes, we devised a model of heat stress in coral embryos (Figure 2). Upon temperature increase, cells respond with regulation of genes playing a role in system perturbation, system maintenance, and system regulation. Heat stress induces differential expression of stress-responsive genes. This in turn results in differential expression of genes involved in the response to oxidative stress as heat stress stimulates the production of ROS [37,38]. High levels of oxidative stress in turn have been linked to programmed cell death pathways and cytoskeletal changes [39]. Furthermore, heat stress causes a general downregulation of metabolic processes in coral embryos. As a consequence, ribosome biosynthesis, metabolism, and oxidative phosphorylation are downregulated. These processes are all interconnected. Additionally, heat stress causes misfolding of proteins, which in turn affects the regulation of the protein degradation machinery. On an upstream level, Histone proteins regulate downstream gene expression, and zinc finger proteins might modify those in turn. Please note that this model is based on incomplete transcriptomic data and manual assortment of differentially expressed genes, and therefore subject to change. Furthermore, due to logistical constraints, we were not able to include true biological replicates. Ideally, three to five independent replicated cultures should have been analyzed per treatment and time point to evaluate the potentially contribution of so-called "jar effects" (i.e. the differences between supposedly identical cultures) into the observed gene expression variation. Although jar effects represent a problem for our analysis, we hope that most of the observed gene expression patterns represent the true response to the treatments, because 1) our results are in correspondence with results from other heat stress studies in other organisms, 2) we find a stronger stress response in larvae treated at higher temperatures, and 3) a similar study that we conducted with *Acropora palmata *larvae in which we did assay biological replicates gave similar results (data not shown). We therefore acknowledge that implications drawn from these data are limited and follow-up studies (e.g. qPCR) with proper biological replication are necessary to validate results and conclusions presented here. Future efforts should concentrate on comparing transcriptomic responses to different stressors in coral embryos in order to identify a set of common and specific key response genes. These key genes could then serve as a repository for stressor-specific biomarkers as well as for research into coral-specific adaptations to stress responses and global climate change.

## Conclusions

Our aim was to look at differential gene expression in coral embryos at temperatures that are known to be non-stressful to corals (27.5°C) in comparison to 1) late summer temperatures during spawning that corals seem to tolerate (29.0°C) and 2) elevated late summer temperatures observed during years when adult bleaching is more pronounced (31.5°C). For this reason, we exposed our embryos to a more constant and gentle increase in water temperature rather than a short term heat shock. Despite the lack of biological replicates in this study, our data indicate that effects of temperature on coral embryos depend on the duration and the degree of temperature change. Embryos at 29.0°C are physiologically responsive and show expression of stress-related genes, but seem to be able to counteract the temperature situation over time. Embryos at 31.5°C, however, are not able to recover during prolonged exposure periods and continuously express stress-related genes.

## Methods

### Sample collection

Gamete bundles containing both egg and sperm of *Montastraea faveolata *were collected from multiple colonies during spawning on the MesoAmerican Barrier Reef in Puerto Morelos, Mexico (20°52'28.77"N and 86°51'04.53"W) on September 3^rd^, 2007. Subsequent embryonic and larval rearing and experimental procedures were carried out at the Unidad de Sistemas Arrecifales of the Instituto de Ciencias del Mar y Limnología (ICMYL) of the Universidad Nacional Autónoma de México (UNAM). Spawn collection and fertilization were carried out at 30.0°C, and followed previously published protocols [40,41]. Two hours after eggs were fertilized, embryos were transferred into bins containing 5 μm filtered seawater (FSW) and were raised at 27.5°C, 29.0°C and 31.5°C. Our temperature exposure system is described in detail in [42] and controlled temperature to to ± 0.2°C. Batch cultures of embryos for this work were contained in 500 mL plastic containers suspended in the temperature baths by a foam rack. Each container was seeded with ca. 5,000 embryos, and embryos for a given temperature were raised in three different containers. Water in the containers was changed twice per day with water that was preheated to the treatment temperature prior to use. Approximately 500 embryos from each of the three containers for a given temperature were sampled and combined after 12 and 48 hours. This gave rise to a single sample of 1,500 embryos for each temperature and time point that was used for subsequent RNA isolation. Each of these RNA isolations was used for 3 technical microarray replicates. Embryos were immediately transferred into RNAlater (Ambion), and incubated overnight in the refrigerator. Subsequently, soaked embryos were frozen at -80°C and returned to the laboratory for analysis. To examine the time course of development, approximately 100 embryos were sampled from each temperature every 4 to 12 hours along the developmental time course. Embryos were sampled from the 500 mL culture containers from the same embryo pool as the microarray samples. Embryos were fixed in 2% Glutaraldehyde in 0.05 M sodium cacodylate buffer and kept at 4°C. The samples were then returned to the laboratory for microscopic examination to determine developmental stages. Experimental research followed internationally recognized guidelines according to CITES (the Convention on International Trade in Endangered Species of Wild Fauna and Flora), permit number: MX-HR-007-MEX. No ethical approval was required for any of the experimental research described here.

### RNA, Hybridization, Microarrays

Microarray protocols followed those established by the Center for Advanced Technology at the University of California, San Francisco http://cat.ucsf.edu/. 1,314 PCR-amplified cDNAs from *M. faveolata *were spotted in duplicate on poly-L-lysine-coated slides yielding a microarray with 2,628 total features. Spotted cDNAs were chosen from EST libraries described in Schwarz *et al*. [43]. 43% of the 1,314 cDNAs had functional annotations as determined by tBLASTx and BLASTx analyses (E-value cut-off 1e-5) against the GenBank non-redundant DNA and protein databases (nr). All clones are accessible via our database at http://sequoia.ucmerced.edu/SymBioSys/index.php.Total RNA of approximately 1,500 frozen coral embryos was isolated using Qiazol lysis reagent (QIAGEN) according to manufacturer's instructions. Embryos were homogenized for 2 minutes using a Mini-Beadbeater (Biospec) with 0.1 mm and 0.55 mm silica beads to break up cellular structures. RNA pellets were cleaned further with RNeasy Mini columns (Qiagen). RNA quantity and integrity was assessed with a NanoDrop ND-1000 spectrophotometer and an Agilent 2100 Bioanalyzer, respectively. For all experiments, 1 μg of total RNA was amplified using the MessageAmp II aRNA kit (Ambion). For cDNA synthesis, 3 μg of aRNA per sample were primed with 3.5 nmoles of random pentadecamers for 10 minutes at 70°C. Reverse transcription (RT) lasted for 2 hours at 50°C using a master mix containing a 4:1 ratio of aminoallyl-dUTP to TTP. Following RT, single-stranded RNA was hydrolyzed by incubating RT reactions in 10 μL 0.5 M EDTA and 10 μL 1 M NaOH for 15 minutes at 65°C. After hydrolysis, RT reactions were cleaned using the MinElute Cleanup kit (Qiagen). Cy3 and Cy5 dyes (GE Healthcare) were dissolved in 18 μL DMSO, and the coupling reactions lasted for 2 hours at room temperature in the dark. Dye-coupled cDNAs were cleaned using the MinElute Cleanup kit (Qiagen). Prior to hybridization, microarrays were post-processed by: 1) UV crosslinking at 60 mJ; 2) a "shampoo" treatment (3 × SSC, 0.2% SDS at 65°C); 3) blocking with 5.5 g succinic anhydride dissolved in 335 mL 1-methyl-2-pyrrilidinone and 15 mL sodium borate; and 4) drying via centrifugation. Appropriate Cy3 and Cy5 labeled cDNAs were mixed together in a hybridization buffer containing 0.25% SDS, 25 mM HEPES, and 3 × SSC. The hybridization mixtures were boiled for 2 minutes at 99°C, then allowed to cool at room temperature for 5 minutes. The cooled hybridization mixtures were pipetted under an mSeries Lifterslip (Erie Scientific), and hybridization took place in Corning hybridization chambers overnight at 63°C. Microarrays were washed twice in 0.6 × SSC and 0.01% SDS followed by a rinse in 0.06 × SSC and dried via centrifugation. Slides were immediately scanned using an Axon 4000B scanner. The experimental setup followed a reference design, i.e. all samples were hybridized against the same pool made up of equal amounts of RNA from all samples. We used three technical replicates for each temperature. Common reference samples were labelled with Cy3, temperature treatment samples with Cy5.

### Data analysis

For the microarrays, slides were scanned as described in [6]. Spot intensities were extracted and background was subtracted using TIGR Spotfinder 2.2.4 [44]. Data was normalized using printtip-specific LOWESS in TIGR MIDAS 2.19 [44]. Data have been deposited in NCBI's Gene Expression Omnibus [45] and are accessible through GEO Series accession number GSE15088. The ratio between the fluorescence intensity of the two channels was then used as input for BAGEL (Bayesian Analysis of Gene Expression Levels) [46]. This analysis yielded relative expression level estimates for 1,218 genes. We used the conservative gene-by-gene criterion of non-overlapping 95% credible intervals to regard a gene as significantly differentially expressed. Genes were defined as up- or downregulated in regard to the expression of that gene in the 27.5°C temperature sample. Fold-changes were calculated as the ratio of the higher expression level to the lower expression level for the temperature conditions to be compared. Significant genes were assorted into categories according to their respective biological processes, cellular components, or manually defined categories. Putative functions were based on GO molecular functions, or manual assessment through literature searches and perusal of protein databases (e.g. PFAM and InterPro). Transcriptome trees were constructed by hierarchical clustering of arrays by the average linkage algorithm on the ratio of the relative expression level as estimated by BAGEL in TIGR TMeV 3.1 [44]. TreeView [47] was used to display the tree.

## Authors' contributions

CRV wrote the manuscript, designed the microarray study, and analyzed the microarray data. JS carried out microarray hybridizations, data extraction and analyses, and wrote the manuscript. LP assisted in microarray data analysis. CJR and AMS raised and provided coral embryos. AMS conceived of the study, and participated in its design and coordination. MM participated in study design, coordination, and wrote the manuscript. All authors read and approved the final manuscript.

## Supplementary Material

Additional file 1 **Differentially expressed genes as determined by BAGEL**. Fold changes are determined in relation to the expression of a gene at 27.5°C. Functional annotation as deduced from BLASTx, GO, and UniProtKB. All CloneIDs are accessible at http://sequoia.ucmerced.edu/SymBioSys/index.php. Click here for file

Additional file 2 **Differentially expressed genes assorted to the three main groups system perturbation, system maintenance, and system regulation**. FC: fold change. All CloneIDs are accessible at http://sequoia.ucmerced.edu/SymBioSys/index.php. Click here for file
